# Combined podocyte, fibrotic and echocardiographic correlates of an operationally defined subclinical cardiorenal phenotype in chronic kidney disease: an exploratory study

**DOI:** 10.1080/0886022X.2026.2698902

**Published:** 2026-07-13

**Authors:** Nilufar Akhmedova, Dostonbek Tukhtaev, Bakhodir Narziev, Akmal Ismoilov, Maftuna Juraeva, Gulomjon Kholov, Ulugbek Ochilov

**Affiliations:** a Bukhara State Medical Institute, Bukhara, Uzbekistan; b Neomed Hospital Private Clinic, Tashkent, Uzbekistan; c Bukhara Branch of the Republican Scientific Center Medical Care, Bukhara, Uzbekistan; d Bukhara State University, Bukhara, Uzbekistan; e Asia International University, Bukhara, Uzbekistan

**Keywords:** Cardiorenal syndrome, chronic kidney disease, nephrinuria, galectin-3, NT-proBNP, echocardiography

## Abstract

Although cardiovascular complications are the most common cause of morbidity and mortality in chronic kidney disease (CKD), the detection of subclinical cardiorenal remodeling is still limited. This is a single-center, exploratory observational study of 127 patients with CKD of various etiologies (hypertensive nephropathy, diabetic nephropathy and chronic glomerulonephritis) and 30 healthy controls. In addition to transthoracic echocardiography at rest and after exercise, biomarkers of podocyte injury (urinary nephrin), fibrosis (urinary type IV collagen and serum galectin-3), cardiac wall stress (serum NT-proBNP) and renal filtration/cardiovascular risk integration (serum cystatin C) were assessed. Biomarker abnormalities were consistent across all CKD groups, even in the absence of overt heart-failure symptoms and preserved ejection fraction. Urinary nephrin and type IV collagen were significantly increased, as were galectin-3 and NT-proBNP concentrations. All CKD groups had resting *E/e′* values above the normal range and these values were further elevated following exercise. Nephrin showed a strong positive correlation with *E/e′* and post-exercise echocardiography was associated with greater discrimination of an operationally defined subclinical cardiorenal phenotype than resting imaging. These exploratory findings indicate that a combined biomarker and echocardiographic profile is associated with an early cardiorenal phenotype and may assist in risk stratification of patients with CKD exhibiting early cardiorenal remodeling prior to the development of overt heart failure; they are hypothesis-generating and not intended to set diagnostic criteria. Prior to implementing this multimarker strategy clinically, larger, multicenter prospective studies with multivariable adjustment and external validation should be performed.

## 1. Introduction

For over 15 years, the bidirectional clinical traffic between the failing heart and the failing kidney has been formalized as the concept of cardiorenal syndrome (CRS) [1–4] and cardiovascular disease is the leading cause of premature death in patients with chronic kidney disease (CKD) [5–7]. However, despite the various consensus statements, integrated risk-prediction models and a growing biomarker toolbox [8–10], cardiology and nephrology practice still tends to identify CRS only after the onset of heart-failure symptoms or when estimated glomerular filtration rate (eGFR) drops below traditional thresholds. At this point, a majority of the preventable damage has been done.

Two parallel observations shape the rationale for the present work. First, large epidemiological surveys and registry analyses converge on a sobering finding: cardiovascular events drive a disproportionate share of the morbidity in CKD and the relative risk increase is already visible at preserved or only mildly reduced eGFR, where overt cardiac symptoms are absent and standard imaging is often unremarkable [5–7,9]. Second, recent mechanistic studies have revealed that CRS is not just a hemodynamic event between two organs but rather a shared molecular pathway, with podocyte injury [11], endothelial dysfunction, profibrotic Wnt/β-catenin and TGF-β signaling [12–14], neurohormonal activation [10,15] and low-grade chronic inflammation [16,17] all acting on the glomerulus, the tubulointerstitium and the myocardium. From this point of view, a marker that only reflects one compartment (eGFR for filtration, NT-proBNP for wall stress, albuminuria for glomerular permeability) is unlikely to capture the syndrome at the time when it is most modifiable.

There are a number of individual biomarkers that have, however, been informative. Podocyte foot-process effacement is accompanied by the appearance of urinary nephrin, a transmembrane protein of the slit diaphragm, which can occur before the onset of albuminuria [11,18]. Type IV collagen, which is the predominant component of the glomerular basement membrane, is a marker of active matrix remodeling and not chronic scarring [16,19]. Galectin-3 has become a clinically relevant marker of myocardial fibrosis and inflammation and has been shown to be prognostic throughout the heart-failure spectrum [20–23]. Although the specificity of NT-proBNP is less in CKD, where both impaired clearance and intracardiac volume increase it, NT-proBNP remains the most operationally useful marker of cardiac wall stress [20,23,24]. Cystatin C is unique in that it is at the crossroads of cardiac and renal physiology and has been associated with both filtration loss and poor cardiac structure [24,25]. None of these markers alone is specific for subclinical CRS and it is the relationships between these markers and to early echocardiographic remodeling that an integrated screening strategy must integrate [18,26,27].

In imaging, the transthoracic echocardiography provides an opportunity in parallel. The ratio of early diastolic transmitral flow velocity to early diastolic mitral-annular velocity (resting *E/e′*) is now known to be a noninvasive surrogate of left-ventricular filling pressure and increases before ejection fraction decreases [28]. However, in CKD, resting echocardiograms in asymptomatic patients are often unremarkable and exercise echocardiograms reveal a much higher proportion of patients with diastolic abnormality [28–31]. In this context, left-atrial volume and post-exercise *E/e′* have proven to be particularly informative [31,32].

However, there are three gaps in this increasing evidence. First, there is no established multimarker approach that integrates podocyte injury, fibrotic activity and echocardiographic remodeling in patients with CKD who have not yet developed heart-failure symptoms [18,24,26]. Second, most published cohorts have been based on advanced CKD or on known HF and the early, modifiable stage is undercharacterized [20,33,34]. Thirdly, there is a lack of comparative studies between the three most common etiologies of CKD in clinical practice: hypertensive nephropathy, diabetic nephropathy and chronic glomerulonephritis, despite the fact that the early molecular pathways of these conditions are likely to be different [11,33,35].

We thus designed an exploratory, prospective cross-sectional biomarker–imaging study with three related aims. The first was to explore, in patients with CKD of three different etiologies and without clinical heart failure, the integrated biomarker profile of urinary nephrin and type IV collagen, serum galectin-3 and NT-proBNP, cystatin C and resting and post-exercise echocardiographic parameters. The second was to investigate whether *E/e′* (interpreted in the context of this multimarker profile) is associated with and may assist in characterizing, an operationally defined early cardiorenal phenotype. The third was to suggest, as a conceptual framework for hypothesis generation, a step-wise approach that could be used to guide future research based on these observations. The study was not intended to define diagnostic criteria for cardiorenal syndrome, but rather to test the working hypothesis that markers of podocyte injury and tissue fibrosis would correlate with cardiac wall-stress and diastolic indices prior to the development of symptomatic heart failure and that stress echocardiography would be associated with greater discrimination of this research-defined phenotype than resting imaging.

## 2. Methods

### 2.1. Study design and setting

This was an exploratory, single-center, two-phase observational study (retrospective audit phase and prospective cross-sectional phase) performed at the Republican Specialized Scientific-Practical Medical Center of Cardiology, Bukhara Regional Branch, Uzbekistan and the Nephrology Department of the Bukhara Regional Multidisciplinary Medical Center, Uzbekistan. In summary, the retrospective phase (2020–2023) described the burden of renal involvement in the spectrum of heart failure in hospitalized patients to provide clinical context, and the prospective phase (March 2023–December 2024) included outpatients and inpatients with CKD and healthy controls, with biomarkers and resting/post-exercise echocardiography performed at a single assessment. In the current analysis, no follow-up of outcomes was performed longitudinally.

The retrospective phase of the study included a retrospective analysis of the medical records of 110 patients treated between 2020 and 2023 at the Bukhara Regional Branch of the Republican Specialized Scientific and Practical Medical Center of Cardiology.

The prospective study was conducted from March 2023 to December 2024 and included 127 patients with arterial hypertension, diabetic nephropathy and chronic glomerulonephritis recruited from the Republican Specialized Scientific and Practical Medical Center of Cardiology and the Nephrology Department of the Bukhara Regional Multidisciplinary Medical Center.

Study flow. In the prospective phase, consecutive eligible patients with CKD of one of the three etiologies and age-matched healthy volunteers, underwent at a single visit: (i) structured clinical and anthropometric assessment; (ii) fasting venous blood sampling and early-morning urine collection for the biomarker panel and renal function; and (iii) resting transthoracic echocardiography followed immediately by post-exercise (six-minute walk test) echocardiography. No fixed exposure or observation period was defined other than this one cross-sectional assessment, and the term “observation period” is used to refer to the enrollment period (March 2023 to December 2024) and not to longitudinal follow-up. The retrospective phase utilized routinely collected patient data from patients treated from 2020 to 2023 and did not require any additional study procedures.

Endpoints. The main outcome was the presence of an operationally defined subclinical cardiorenal phenotype (as described in Section 2.7). Secondary endpoints were exploratory and included: (i) associations (correlations) between the renal, fibrotic and cardiac biomarkers; (ii) associations between these biomarkers and resting and post-exercise echocardiographic indices, primarily E/e′; and (iii) discriminative association of resting and post-exercise *E/e′* with the operationally defined phenotype relative to healthy controls. These endpoints were exploratory and not designed to define or confirm the diagnosis of cardiorenal syndrome.

The study was carried out in compliance with the Declaration of Helsinki (revised in Seoul, 2008) and approved by the Institutional Ethics Committee of Abu Ali ibn Sino Bukhara State Medical Institute. All participants provided written informed consent.

### 2.2. Retrospective cohort

The records of 110 hospitalized patients with known CHF (NYHA functional class and confirmed by 6-min walk test) were analyzed for the presence of renal dysfunction (eGFR using the CKD-EPI creatinine equation and KDIGO 2020 staging). The aim of this phase was to describe the prevalence and clinical associations of renal involvement throughout the spectrum of heart failure severity, to give a denominator for the prospective findings.

### 2.3. Prospective cohort

We recruited 127 consecutive patients with CKD of three etiologies attending outpatient and inpatient services. There were 52 patients in group 1 with hypertensive nephropathy, 41 patients in group 2 with type 2 diabetic nephropathy and 34 patients in group 3 with biopsy proven chronic glomerulonephritis. Inclusion criteria included (i) age *≥ 18* years, (ii) for hypertensive and diabetic nephropathy, disease duration ≤ 5 years to enrich for early disease, (iii) for chronic glomerulonephritis, disease duration ≤ 10 years, (iv) absence of clinical heart-failure symptoms and signs at enrollment and (v) ability to perform an exercise echocardiogram. Ischemic heart disease, previous myocardial infarction, clinically significant arrhythmia, acute decompensation, renal replacement therapy, severe non-cardiac comorbidity and pregnancy were all exclusion criteria. The control group consisted of 30 age matched healthy volunteers without hypertension, diabetes, known kidney disease or cardiovascular medication obtained from periodic health screening.

### 2.4. Clinical and biochemical assessment

All participants underwent clinical and biochemical assessments. All participants were subjected to a structured clinical evaluation which included history, anthropometry, blood pressure and routine biochemistry. Venous blood was sampled in the morning after an 8–14 h fast. A Mindray BA-88 analyzer was used for complete blood count and for biochemistry (urea, creatinine, glucose, glycated hemoglobin, lipid panel, electrolytes) Human (Germany) reagents were used. Serum creatinine was determined using a Jaffé method and urea was determined by colorimetric method. Estimated glomerular filtration rate (eGFR) was calculated using both serum creatinine and cystatin C using the CKD-EPI 2021 equations and CKD staging was done according to KDIGO criteria. After protein denaturation with bezethonium-chloride, 24-h albuminuria was photometrically measured and staged according to KDIGO 2020. Concomitant cardiovascular and renoprotective medication (ACE inhibitors/ARBs, SGLT2 inhibitors, diuretics, beta-blockers and statins) were not systematically recorded in this exploratory cohort, and the implications for confounding and for the proposed analyses are discussed in the Limitations.

### 2.5. Cardiorenal biomarkers

The serum level of cystatin C was determined by immunoturbidimetry (DiaSys, Germany, range 0.58–1.02 mg/L). In a subset, a renal functional reserve test was performed by re-measuring cystatin C 90 min after a standardized protein load (1 g/kg unsalted egg white).

Urinary nephrin was determined by sandwich ELISA (DiMediTec Diagnostics, Germany) according to the manufacturer’s instructions on midstream early-morning urine, which was centrifuged and stored at *≤ −20 °C*. Urinary type IV collagen was measured by sandwich ELISA with a calibration range of 7.8–500 ng/mL; absorbance was measured at 450 nm using a microplate reader.

Serum galectin-3 was measured using a commercial ELISA kit (human Galectin-3 ELISA, Germany; analytical sensitivity 0.29 ng/mL). The reference range was 3.7–11.7 ng/mL. Serum NT-proBNP was assayed on the same platform, in accordance with standard pre-analytical recommendations (overnight fast, single venepuncture, immediate centrifugation). The NT-proBNP test was not used as a single diagnostic criterion but as an additional marker, as is currently done, because of its limited specificity in advanced renal dysfunction.

### 2.6. Echocardiography

All echocardiograms were performed on a Cetus *40* Focus & Fusion system (China) following American Society of Echocardiography recommendations. *M*- and *B-mode* imaging in parasternal long-axis, parasternal short-axis and apical four-chamber views yielded left-ventricular end-diastolic and end-systolic dimensions and volumes, posterior-wall and interventricular-septal thicknesses and right- and left-atrial dimensions and stroke volume. Left-ventricular mass was calculated using the Devereux formula and using body-surface area, left-ventricular hypertrophy was defined as *≥ 115 g/m^2^* in men and *≥ 95 g/m^2^* in women. Four geometry categories were identified based on relative wall thickness according to A. Ganau’s nomenclature.

Diastolic function was defined by pulsed-wave transmitral inflow (*E, A, E/*A ratio, deceleration time, isovolumic relaxation time) and tissue-Doppler interrogation of septal and lateral mitral-annular velocities *(e′)*, the average of which was used to calculate *E/e′*. The locally accepted upper normal value of *E/e′* was 14. Three diastolic-dysfunction patterns were defined according to the ASE/EACVI recommendations: impaired relaxation, pseudonormal and restrictive.

The physiological stressor was a six-minute walk test and stress echocardiography was performed immediately after the walk test [28,31]. Besides the changes in *E*, *e′* and *E/e′,* special attention was given to the left-atrial volume index, which we thought to be a sensitive marker of subclinical remodeling [32]. All prospective participants underwent a complete echocardiographic protocol (resting and post-exercise).

With respect to the post-exercise protocol, resting images were acquired first; the patient then performed a standardized six-minute walk test and post-exercise images were acquired immediately on return to the examination couch, with the diastolic indices (*E, e′* and *E/e′*) captured as a priority within the first few minutes after exercise to limit recovery-related attenuation of the load-dependent signal. To minimize variability in measurements, all studies were conducted and analyzed by experienced operators from the cardiology service, and the same machine was used for all studies, with the same acquisition parameters for resting and post-exercise images. However, we recognize that formal measures of inter- and intra-observer reproducibility (e.g., intraclass correlation coefficients or coefficients of variation) and a precisely standardized post-exercise acquisition window were not prospectively collected in this exploratory study, and are planned for any future multi-center extension, as a limitation.

### 2.7. Outcome definitions

The primary endpoint of the prospective analysis was a research-based, operationally defined subclinical cardiorenal phenotype defined as the presence of (i) any CKD etiology with at least KDIGO stage A2 albuminuria or eGFR < 90 mL/min/1.73 m^2^ and (ii) at least one echocardiographic parameter outside the normal range, most often *E/e′* > 14 at rest or > 14 after exercise, in the absence of symptomatic heart failure. The threshold for comparison with controls was chosen *a priori* on physiological grounds and not by statistical optimization to avoid circular performance estimates. This composite is an operational research definition, and is not synonymous with clinical diagnosis of cardiorenal syndrome.

We should make it clear that there is no universally accepted, internationally standardized definition of subclinical cardiorenal syndrome at present. For the purposes of this exploratory study, we used the operational research definition of the term above, as there was no consensus definition. This definition was operationally derived by combining a well-known marker of renal involvement (KDIGO albuminuria/eGFR category) with an objective echocardiographic marker of elevated left-ventricular filling pressure (E/e′) in patients without overt heart failure, and was not derived from or validated against an external reference standard. Therefore, the resulting phenotype should be viewed as a research-defined construct that needs to be externally validated in independent cohorts prior to any clinical use and all findings related to it should be interpreted as exploratory and hypothesis-generating.

### 2.8. Statistical analysis

Continuous variables are presented as mean ± standard error and discrete variables as counts and percentages. The Student t-test was used for normally distributed variables, the Mann–Whitney U test for other variables and the χ^2^ test or Fisher’s exact test for categorical variables for between-group comparisons. Trends across CKD etiologies or heart-failure functional classes used analysis of variance with post-hoc adjustment. Spearman coefficient was used to quantify the correlations between continuous variables. Two-sided p-values *< 0.05* were considered significant; in tables we mark *p* < 0.05, < 0.01 and < 0.001 by *, ** and ***, respectively. Receiver-operating-characteristic analysis was used to describe the discriminative association of *E/e′* with the operationally defined subclinical cardiorenal phenotype, with area-under-the-curve (AUC) and 95% confidence intervals (CI); odds ratios are provided for each AUC estimate. No imputation was done; analyses were complete-case. Analyses were performed using standard biomedical statistical software. Given the exploratory nature of the study, several aspects of the statistical approach should be made explicit. First, only unadjusted between-group comparisons and bivariate (Spearman) correlations were performed, and no multivariable regression modeling was done to adjust for potential confounders (age, sex, eGFR, albuminuria, blood pressure, diabetes status or concomitant medication). Multivariable adjustment would be preferable, but the sample size and event structure were not thought to be adequate to develop a stable multivariable predictive model that includes all clinically relevant covariates. The present results should thus be viewed as associations and not independent effects of the predictors. The data set was created for descriptive and correlation/ROC analyses and was not designed or powered to allow for robust multivariable adjustment, and the associations reported should not be interpreted as independent of these factors and residual confounding cannot be ruled out. Second, because numerous biomarker and group comparisons were undertaken, no formal correction for multiple comparisons (for example, Bonferroni or false-discovery-rate methods) was applied; the reported p-values are therefore nominal, the analyses are exploratory and the possibility of type I error (false-positive associations) is increased. Third, no formal *a priori* sample-size or statistical-power calculation was done, and the sample size was determined by the number of patients available during the enrollment window, which may have been underpowered for some comparisons. These options are revisited in the Limitations section.

## 3. Results

### 3.1. Cohort characteristics

The three CKD groups and the control arm were generally similar in age but the chronic-glomerulonephritis group was slightly younger and had a longer duration of disease (*9.1 ± 1.1* years) than the other two groups (*4.7–4.8* years for hypertensive and diabetic nephropathy). As expected, the body-mass index, systolic and diastolic blood pressure, fasting glucose and lipid panel showed the expected distribution across CKD etiologies, with the highest blood-pressure burden in the hypertensive nephropathy group (systolic *155.3 ± 3.5 mm Hg*), the highest fasting glucose in the diabetic-nephropathy group (*7.8 ± 0.9 mmol/L*) and the most marked renal biochemical disturbance in the glomerulonephritis group (urea *19.2 ± 1.8 mmol/L*, creatinine *229.4 ± 18.8 µmol/L*). The baseline demographic, clinical and laboratory characteristics of the study population are summarized in Table 1. However, none of the potential participants had symptomatic heart failure at enrollment.

**Table 1.** Baseline clinical and laboratory characteristics of the prospective cohort.

Variable	Control	Group 1: HN	Group 2: DN	Group 3: CGN	p*
n	30	52	41	34	—
Age, years	48.1 ± 1.4	52.4 ± 1.5	50.3 ± 1.8	44.7 ± 2.1	<0.05
Male, %	53.3	40.4	63.4	50.0	<0.05
Disease duration, years	—	4.8 ± 0.5	4.7 ± 0.8	9.1 ± 1.1	<0.01
BMI, kg/m²	25.7 ± 1.0	27.7 ± 0.7	29.7 ± 0.9	26.4 ± 0.8	<0.05
Systolic BP, mm Hg	117.8 ± 4.2	155.3 ± 3.5	141.3 ± 4.5	138.3 ± 3.7	<0.05
Diastolic BP, mm Hg	78.4 ± 3.4	113.0 ± 1.2	92.4 ± 2.2	91.7 ± 2.4	<0.05
Fasting glucose, mmol/L	4.7 ± 0.3	5.8 ± 0.8	7.8 ± 0.9	5.6 ± 0.4	<0.05
Total cholesterol, mmol/L	4.9 ± 1.1	5.9 ± 0.7	6.2 ± 0.8	6.2 ± 0.9	<0.05
Triglycerides, mmol/L	1.07 ± 0.24	1.87 ± 0.6	2.20 ± 0.7	1.80 ± 0.9	<0.05
LDL-C, mmol/L	3.3 ± 0.9	4.8 ± 0.6	4.5 ± 0.7	4.7 ± 0.8	<0.05
HDL-C, mmol/L	1.5 ± 0.2	1.02 ± 0.4	1.08 ± 0.5	1.26 ± 0.6	<0.01
Urea, mmol/L	3.7 ± 0.9	8.7 ± 0.7	8.4 ± 0.8	19.2 ± 1.8	<0.01
Creatinine, µmol/L	84.1 ± 2.9	109.3 ± 7.2	104.9 ± 8.1	229.4 ± 18.8	<0.01

*Note.* Values are presented as mean ± standard error or proportion (%). *P values indicate comparisons of each CKD group with the control group.

Abbreviations: BMI, body-mass index; BP, blood pressure; CGN, chronic glomerulonephritis; DN, diabetic nephropathy; HDL-C, high-density-lipoprotein cholesterol; HN, hypertensive nephropathy; LDL-C, low-density-lipoprotein cholesterol.

### 3.2. Cardiorenal involvement retrospective burden

Of the 110 patients with heart failure who were hospitalized in the retrospective audit, 22 were NYHA functional class I, 41 were class II and 47 were class III. Most (22.5% with CKD stage 1, 36.7% with CKD stage 2 and 32.4% with CKD stage 3a–b) had CKD and the prevalence of more advanced CKD increased monotonically with heart-failure functional class. Albuminuria increased significantly with increasing symptoms (mean A-stage *148 mg/g* in NYHA I, *307 mg/g* in NYHA III; *p < 0.001*) and NT-proBNP increased almost twofold from class I to class III. These findings were consistent, in our population, with the view that the cardiorenal continuum is not a rare association but rather the default phenotype in advanced heart failure [1,5,9,20] and prompted the prospective question: when is it detectable along the kidney-disease trajectory?

### 3.3. Renal and cardiac biomarkers

The four principal biomarkers demonstrated progressive elevations across CKD etiologies across the prospective cohort. Galectin-3 levels were higher in hypertensive nephropathy (11.4 ± 0.7 ng/mL, *p* < 0.01 versus control), diabetic nephropathy (13.6 ± 0.8 ng/mL, *p* < 0.01 versus control) and chronic glomerulonephritis (15.6 ± 0.83 ng/mL, *p* < 0.01 versus control) compared to controls, with a trend of increasing levels with disease severity (trend *p* < 0.001). NT-proBNP rose in parallel—from 118.7 ± 1.7 pg/mL in controls to 253.2 ± 1.2, 236.7 ± 0.9 and 301.8 ± 0.8 pg/mL across the three CKD groups (*p* < 0.01 versus control).

**Figure 1.**
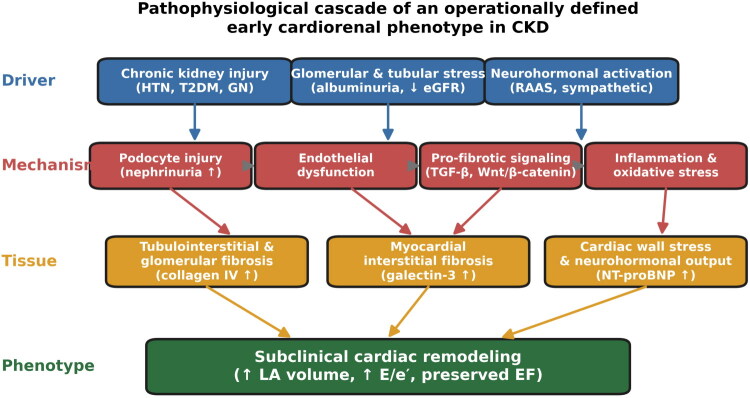
Pathophysiological cascade of an operationally defined early cardiorenal phenotype in CKD. The parallel mechanistic pathways that are activated by upstream renal drivers (hypertension, diabetes, glomerulonephritis) converge on tissue-level remodeling in the kidney (tubulointerstitial fibrosis, urinary nephrin and type IV collagen) and the myocardium (interstitial fibrosis, serum galectin-3; wall stress, serum NT-proBNP). The integrated downstream phenotype is subclinical cardiac remodeling with preserved ejection fraction, enlarged left atrium and increasing E/e′.

The two renal-injury biomarkers showed greater separation between etiologies. Urinary nephrin was slightly increased in hypertensive nephropathy (6.9 ± 0.7 ng/mL, ∼ 4-fold higher than control) and in diabetic nephropathy (7.4 ± 0.5, ∼ 4.3-fold) but was significantly increased in chronic glomerulonephritis (14.1 ± 0.3 ng/mL, ∼ 8-fold higher than control, *p* < 0.01 inter-group). A similar but less steep, gradient was observed for urinary type IV collagen (Table 2). This is in line with the cellular biology of these diseases [11,18]: in the early phase of hypertensive and diabetic nephropathy, filtration and tubulointerstitial integrity are mainly affected, while in chronic glomerulonephritis, podocyte and slit-diaphragm injury are more pronounced and matrix turnover is increased. The pathways of upstream renal injury converge on myocardial fibrosis and wall stress and diastolic dysfunction, which underlie subclinical cardiorenal remodeling in CKD (Figure 1).

**Table 2.** Renal, fibrotic and cardiac biomarkers across CKD etiologies.

Biomarker	Control	HN (G1)	DN (G2)	CGN (G3)
N	30	52	41	34
Galectin-3, ng/mL	8.6 ± 0.9	11.4 ± 0.7*	13.6 ± 0.8**^a^	15.6 ± 0.83**^a^
NT-proBNP, pg/mL	118.7 ± 1.7	253.2 ± 1.2*	236.7 ± 0.9*^a^	301.8 ± 0.8**^b^
Urinary nephrin, ng/mL	1.7 ± 0.9	6.9 ± 0.7**	7.4 ± 0.5**^a^	14.1 ± 0.3**^b^
Urinary type IV collagen, µg/L	21.2 ± 1.3	29.7 ± 1.1*	31.8 ± 0.9*^a^	42.7 ± 0.9**^a^

Values are mean ± standard error.

* *p* < 0.05; **p < 0.01 versus control.

^a^ *p* < 0.05.

^b^ *p* < 0.01 for inter-group comparison.

Abbreviations: CGN, chronic glomerulonephritis; DN, diabetic nephropathy; HN, hypertensive nephropathy.

### 3.4. Inter-marker correlations

**Figure 2.**
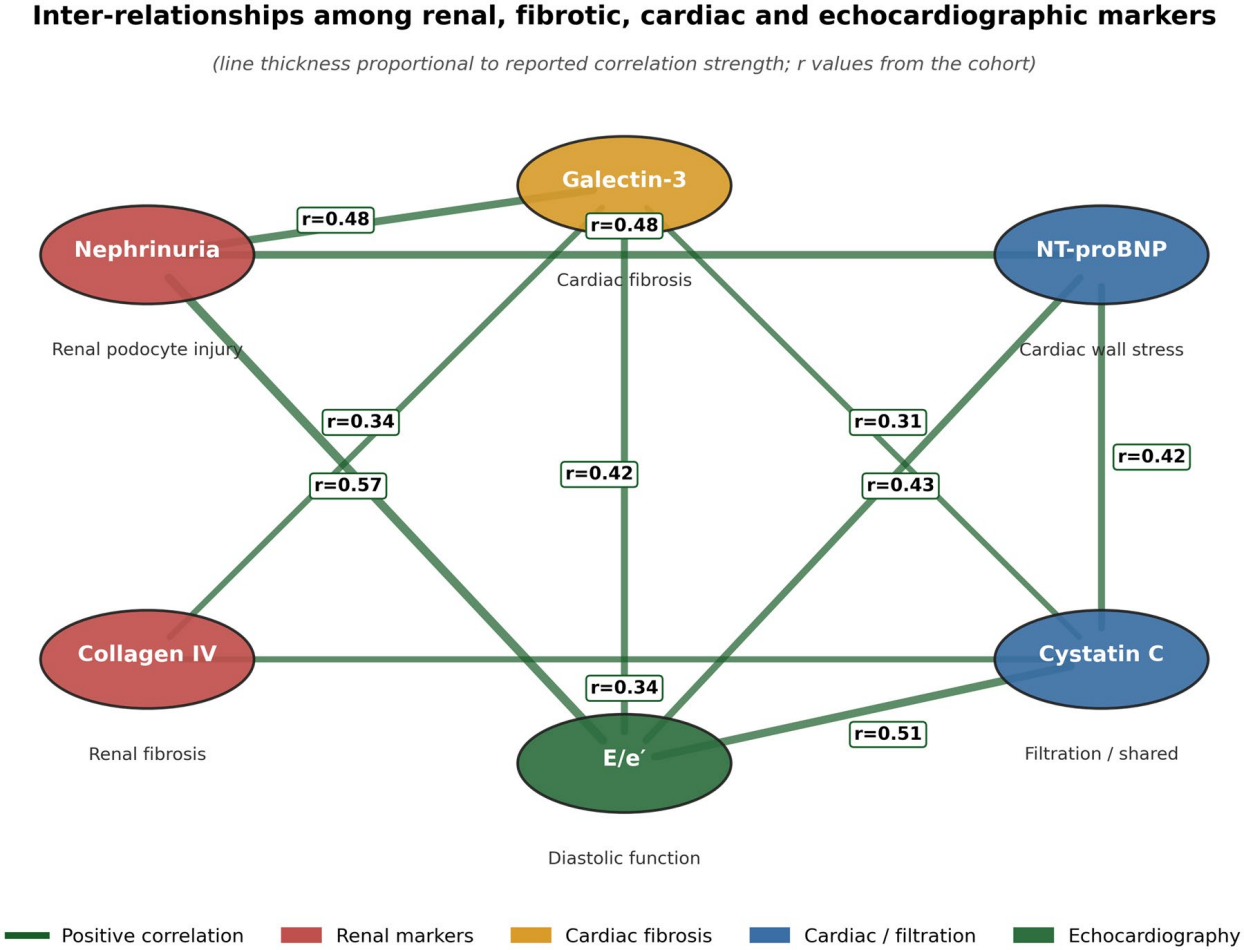
Inter-relationships among renal, fibrotic, cardiac and echocardiographic markers. The thickness of the line is proportional to the absolute value of the Spearman correlation coefficient that is reported in the cohort. Renal markers (urinary nephrin, type IV collagen) are associated with cardiac fibrosis (galectin-3) and cardiac wall-stress (NT-proBNP) markers, both of which converge on the diastolic-function index *E/e′* that combines anatomical and haemodynamic remodeling. The highest correlations are between urinary nephrin and *E/e′* and between cystatin C and *E/e′*, supporting the concept of podocyte injury and dual-compartment filtration markers as upstream markers of subclinical cardiorenal involvement.

The biomarkers were not independent of each other. The strongest renal–cardiac association was the correlation between urinary nephrin and serum NT-proBNP (*r = 0.48, p < 0.01*)—a finding that integrates the cellular biology of the slit diaphragm with the hemodynamics of the left ventricle [11,20]. Urinary nephrin was also significantly correlated with serum galectin-3 (*r = 0.48, p < 0.01*), indicating that podocyte injury and myocardial fibrosis are partially synchronized processes and not independent events [12–14]. The matrix-turnover marker type IV collagen correlated with galectin-3 (*r = 0.34, p < 0.01*), a kidney-fibrosis–heart-fibrosis association with potential biological plausibility [16,19]. As expected for a molecule with a dual kidney–cardiac location, cystatin C correlated with galectin-3 (*r = 0.31, p < 0.01*) and NT-proBNP (*r = 0.42, p < 0.001*). The overall correlation matrix is presented in Figure 2.

### 3.5. Echocardiographic phenotype: resting and post-exercise

Modest but consistent abnormalities were found at resting echocardiography (Table 3). Conventional volumes were not significantly different from controls and left-ventricular ejection fraction was preserved in all groups (54–55%). In all CKD groups, right-ventricular systolic pressure was already increased *(≈ 24–30* mm Hg versus *19* in controls) [36]. The principal resting abnormality involved diastolic function: e′ was sharply reduced (0.06 m/s versus 0.107 in controls) and *E/e′* exceeded the upper-normal threshold of 14 in all the CKD groups (*14.1–14.3* [28]). Left-atrial diameter was mildly increased (*38.2–39.1* mm vs *37.1* mm in controls) [32].

**Table 3.** Resting echocardiographic parameters.

Parameter	Control	HN (G1)	DN (G2)	CGN (G3)
LA diameter, mm	37.1 ± 1.4	38.7 ± 1.0**	38.2 ± 1.2*	39.1 ± 1.4**
RA diameter, mm	37.4 ± 1.3	38.2 ± 0.76*	37.5 ± 1.1*	38.7 ± 1.3*
PASP, mm Hg	18.9 ± 2.6	26.4 ± 2.8**	23.7 ± 2.5*	29.5 ± 3.1**
LV end-systolic volume, mL	61.2 ± 3.2	65.1 ± 1.9*	62.4 ± 2.1*	67.6 ± 2.5**
LV end-diastolic volume, mL	152.4 ± 5.2	150.4 ± 5.2*	143.7 ± 4.6*	153.4 ± 4.8*
LV ejection fraction, %	53.6 ± 1.5	54.5 ± 1.5*	53.6 ± 1.3*	54.7 ± 1.1*
E, m/s	0.56	0.86**	0.87**	0.87**
e′, m/s	0.107	0.06***	0.061***	0.062***
*E/e′*	5.2	14.3***	14.2***	14.1***

* *p* < 0.05; **p < 0.01; ***p < 0.001 versus control.

Abbreviations: LA, left atrium; LV, left ventricle; PASP, pulmonary-artery systolic pressure; RA, right atrium.

Diastolic dysfunction became more apparent following exercise (Table 4). *E/e′* increased to *16.3* in hypertensive nephropathy, *15.1* in diabetic nephropathy and *17.4* in chronic glomerulonephritis (compared with 4.1 in controls; *p < 0.001* for all comparisons). There was also an increase in right-ventricular systolic pressure with exercise, especially in the glomerulonephritis group (*29.5* mm Hg) [36,37]. Importantly, ejection fraction remained preserved (54.5–54.9%), so the abnormality was diastolic and load-sensitive—exactly the substrate that resting echocardiography commonly misses in this population [28,29,38,39].

**Table 4.** Post-exercise echocardiographic parameters.

Parameter	Control	HN (G1)	DN (G2)	CGN (G3)
LV ejection fraction, %	53.6 ± 1.5	54.5 ± 1.5*	54.6 ± 1.3*	54.9 ± 1.1*
PASP, mm Hg	18.9 ± 2.6	26.4 ± 2.8**	23.7 ± 2.5*	29.5 ± 3.1**
LV end-systolic volume, mL	61.2 ± 3.2	68.1 ± 3.3*	62.4 ± 3.0*	70.6 ± 2.5**
E, m/s	0.47	0.85**	0.86**	0.87**
e′, m/s	0.117	0.052***	0.057***	0.05***
*E/e′* post-exercise	4.1	16.3***	15.1***	17.4***

* *p* < 0.05; **p < 0.01; ***p < 0.001 versus control.

Abbreviations: LV, left ventricle; PASP, pulmonary-artery systolic pressure.

### 3.6. Exploratory discriminative association of *E/e′*

**Figure 3.**
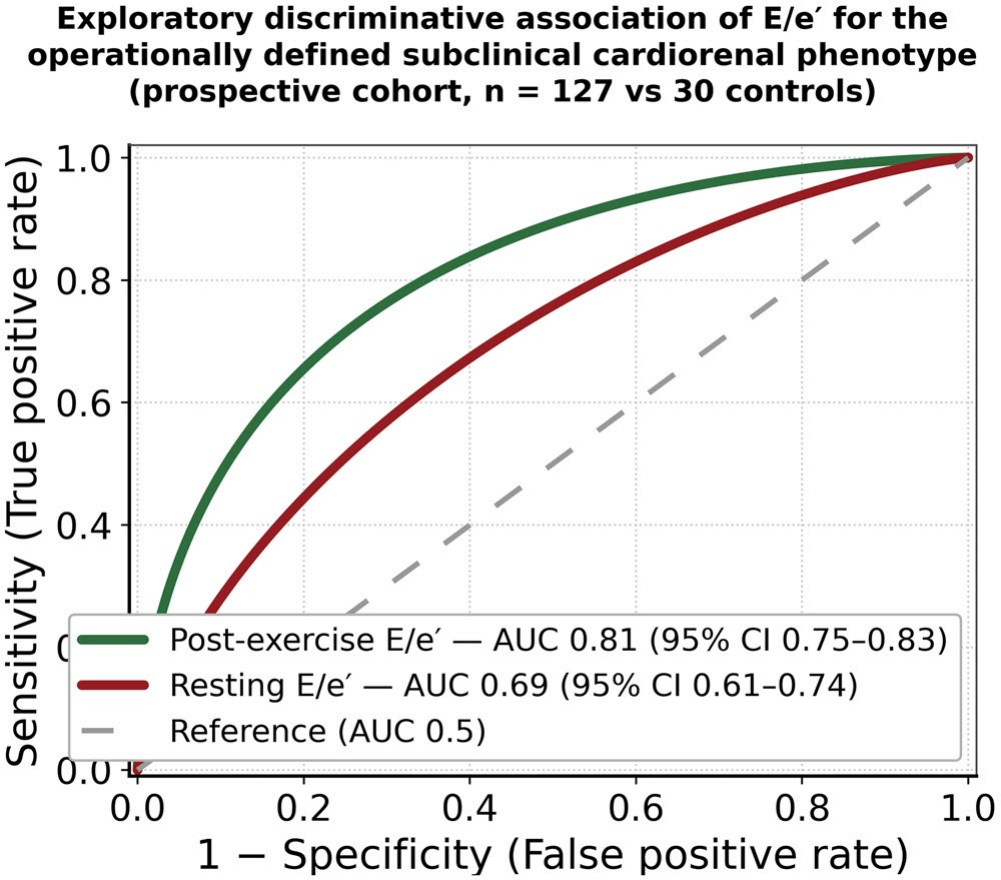
Receiver-operating-characteristic curves for E/e′. Resting *E/e′* (red curve) has an AUC of 0.69 (95% CI 0.61–0.74) for the exploratory discriminative association with the operationally defined subclinical cardiorenal phenotype in CKD patients without overt heart failure. The AUC is numerically higher at 0.81 (0.75–0.83) (green curve) by adding a six-minute walk test as a physiological stressor and re-imaging immediately afterwards. The diagonal dashed line is the non-informative reference.

We then investigated the ability of *E/e′* (as defined operationally in the multimarker context above) to distinguish the operationally defined subclinical cardiorenal phenotype from the healthy control phenotype. Resting *E/e′* was associated with an AUC of 0.69 (95% CI 0.61–0.74; odds ratio 2.1; *p* < 0.01). Post-exercise *E/e′* was associated with a numerically higher exploratory discriminative association with an AUC of 0.81 (0.75–0.83; odds ratio 6.4; *p* < 0.001) (Table 5; Figure 3). This numerically higher discriminative association following exercise was a preliminary exploratory finding that was similar across the three CKD etiologies and was numerically greatest in the glomerulonephritis group, where the resting echocardiogram is most likely to mislead [28,37–39].

**Table 5.** Discriminative association of *E/e′* with the operationally defined subclinical cardiorenal phenotype.

Parameter	AUC	SE	OR	95% CI/*p*
Resting *E/e′*	0.69	0.03	2.1	0.61–0.74; *p* < 0.01
Post-exercise *E/e′*	0.81	0.02	6.4	0.75–0.83; *p* < 0.001

Abbreviations: AUC, area under the receiver-operating-characteristic curve; CI, confidence interval; OR, odds ratio; SE, standard error of the AUC.

### 3.7. Correlations of *E/e′* with the biomarker panel

*E/e′* was significantly correlated with all of the biomarkers. The highest correlation was with urinary nephrin (*r ≈ 0.56* in hypertensive nephropathy, *0.54* in diabetic nephropathy, *0.60* in chronic glomerulonephritis, pooled *r ≈ 0.55, p < 0.001*). Correlations with cystatin C (*r ≈ 0.50–0.52*), galectin-3 (*r ≈ 0.40–0.45*), NT-proBNP (*r ≈ 0.40–0.49*) and type IV collagen (*r ≈ 0.20–0.36*) were all positive and consistent across groups. The correlation with eGFR was, unsurprisingly, negative, with a moderate correlation (*r ≈ −0.45 to −0.51*) indicating that worse filtration was associated with higher diastolic filling pressures [31,32]. This is in line with the concept that *E/e′* is a hemodynamic reflection of the combined renal–fibrotic–cardiac signal and not an echocardiographic parameter alone [19,28].

### 3.8. Conceptual, hypothesis-generating framework

**Figure 4.**
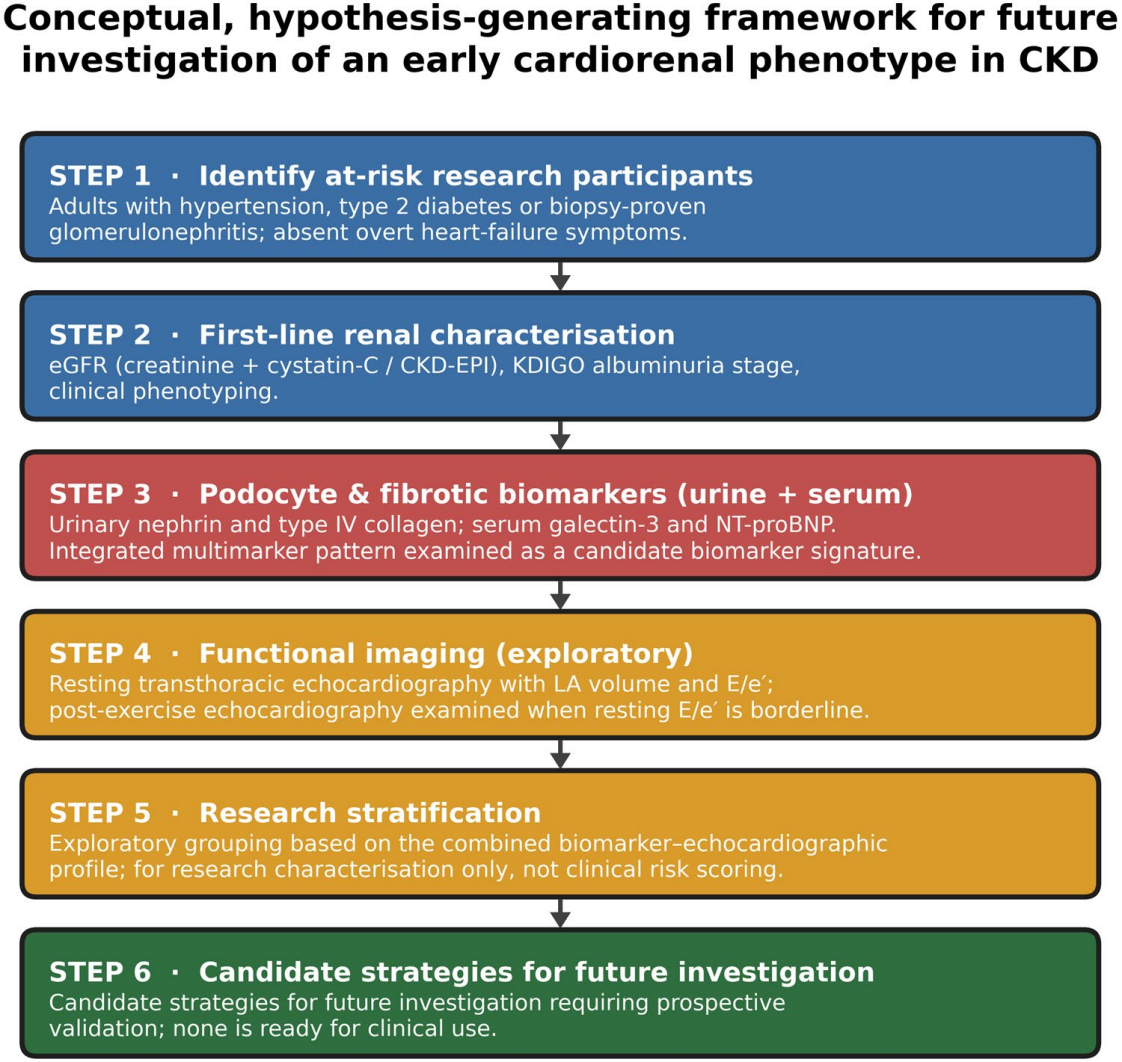
Conceptual framework for future investigation of an early cardiorenal phenotype in CKD (exploratory and hypothesis-generating; not a validated screening algorithm and not recommended for clinical implementation without prospective external validation). A six-step conceptual framework comprising identification of at-risk research participants (step 1), first-line renal characterization (step 2), the podocyte–fibrotic biomarker panel examined as a candidate biomarker signature (step 3), resting and post-exercise echocardiography (step 4), exploratory research stratification based on the combined biomarker–echocardiographic profile (step 5) and candidate strategies for future investigation requiring prospective validation (step 6). The framework is offered as hypothesis-generating only; it is not a clinical decision-support tool or a recommended management pathway and each component needs to be prospectively validated before use in the clinic.

Based on the above observations, we propose a six-step conceptual framework as a hypothesis-generating tool that may, in the future, guide research into early cardiorenal involvement (Figure 4); it is presented as an exploratory model and is not a clinical decision-support tool, a validated screening algorithm or a recommended management pathway. Steps 1–2 are related to identification of at-risk research participants and first-line renal characterization (eGFR, KDIGO albuminuria stage). The multimarker panel (Step 3) includes urinary nephrin and type IV collagen, serum galectin-3 and NT-proBNP, which was evaluated as a potential signal [22–24,26]. Step 4 is resting echocardiography focusing on left-atrial volume index and *E/e′* and post-exercise imaging is considered when resting *E/e′* is borderline [28,32]. Step 5 indicates exploratory research stratification based on the combined biomarker–echocardiographic profile (for research characterization only, not clinical risk scoring) and Step 6 provides a list of candidate pathophysiological frameworks requiring prospective validation, including areas requiring prospective evaluation and candidate risk-modifying strategies. This framework is based on exploratory data from a single center, cross-sectional and unadjusted for clinical use. The proposed framework needs to be validated in independent cohorts prior to clinical use.

## 4. Discussion

Three results are noteworthy. First, in CKD patients without overt heart-failure symptoms, urinary nephrin, urinary type IV collagen, serum galectin-3 and NT-proBNP demonstrate concordant elevations measurable by routine laboratory methods. Second, this multimarker signature is consistent with echocardiographic evidence of diastolic dysfunction, especially with *E/e′* and the correlations are similar for the different etiologies (hypertensive, diabetic and glomerulonephritic). Third, the discriminative power of *E/e′* for the subclinical cardiorenal phenotype increases significantly when imaging is performed after a simple 6-min walk test (AUC *0.69 to 0.81*). These exploratory results support the hypothesis that biomarker signatures associated with early cardiorenal remodeling can be detected prior to clinical presentation and that markers for this purpose are already available in routine assay catalogues. These observations are unadjusted and the phenotype was operationally defined, and thus are hypothesis-generating and need to be confirmed in adjusted, externally validated cohorts.

The mechanistic interpretation is consistent with the current cardio-nephrology literature. Podocyte injury, as reflected by urinary nephrin, is one of the earliest cellular events in glomerular disease and shedding of nephrin into the urine is an early event in experimental and human studies, preceding clinically meaningful albuminuria by hyperglycemia, ischemia and RAAS activation and by mechanical stress [11,18]. Tubulointerstitial fibrosis, indexed here by urinary type IV collagen, is the second major axis: it reflects active matrix turnover rather than chronic scarring and provides a parallel renal-injury readout [16,19]. On the cardiac side, galectin-3 is a profibrotic mediator and a clinical marker of interstitial myocardial remodeling [20–22] and NT-proBNP is a useful integrator of wall stress (although it is not specific in CKD due to impaired clearance) [20,23,24]. The observed associations between these renal and cardiac axes, such as the association between type IV collagen and galectin-3, support the current concept of CRS as a common molecular pathway with TGF-β and Wnt/β-catenin signaling as the core [12–14].

Our findings are consistent with previous literature that has suggested integrated, multi-analyte biomarker strategies in cardiorenal disease over single-marker approaches [21–25,27,34] and the use of early-renal-injury markers in patients at risk of CRS [18,26,40] and the description of shared heart–kidney fibrotic signaling pathways [12–14]. We add direct, prospective, multimarker data in the population for which these frameworks were developed: CKD patients prior to the development of symptomatic heart failure.

The echocardiographic findings deserve particular emphasis. Diastolic dysfunction is well documented in CKD [28] but resting echocardiography often underestimates the severity, particularly in those with preserved ejection fraction [29,30]. Diffuse myocardial fibrosis and subclinical filling-pressure abnormalities have been shown in comprehensive reviews of diastolic function in CKD and cardiac MRI studies, in patients with an apparently normal resting echocardiogram [28,29,38,39]. Our observations are consistent with this literature and support the hypothesis that stress echocardiography may be useful in CKD, where it is underutilized [28,31,37]. This difference in discrimination may be relevant: an odds ratio of 6.4 for post-exercise *E/e′* is a larger exploratory discriminative association than the resting odds ratio of 2.1.

The observed biomarker profile is biologically plausible and depends on the etiology. In chronic glomerulonephritis, the immune-mediated damage directly affects the glomerular tuft and urinary nephrin and type IV collagen increased most rapidly in this group, with downstream cardiac fibrosis markers (galectin-3 and NT-proBNP) increasing in parallel [11,18]. In the first five years of disease, the renal signal was more modest, as the filtration loss has not yet stimulated the matrix turnover to the same extent but the cardiac signals were still detectable and correlated with the renal markers [15,20,33] in the case of hypertensive and diabetic nephropathy. This trend is similar to previous findings of an albuminuria–cardiac-phenotype relationship in CKD populations [20,31,32] and myocardial structural abnormalities throughout the spectrum of CKD using cardiac magnetic resonance [38,39]. From a clinical perspective, the message is that an integrated panel is useful irrespective of etiology but the relative importance of each marker will differ and should be considered in the context of the renal diagnosis.

There are several new molecular pathways that could be relevant to the renal–cardiac signal seen here and are only mentioned as potential avenues for future research [35,41,42].

Lastly, a low-cost urine and serum biomarker panel with stress echocardiography may be an appealing alternative in resource-limited areas where cardiac MRI is not readily available [43,44].

### 4.1. Clinical implications

The main potential implication of these exploratory findings is that a subclinical cardiorenal phenotype in CKD could be detected with widely available tests, which would require validation prior to clinical application. Urinary nephrin and type IV collagen ELISAs, serum galectin-3 and NT-proBNP immunoassays, cystatin-C-based eGFR and resting plus post-exercise echocardiography are, in principle, available in a reasonably equipped cardio-nephrology service [22,24,26]. Such early detection, if confirmed, could in theory be used to redirect patients to optimized blood-pressure control, glycemic management, RAAS-blockade and SGLT2-inhibition before the onset of structural heart failure [6,10,15] but our cross-sectional data cannot prove any effect on outcomes. The recently proposed cardiovascular-kidney-metabolic framework of the American Heart Association is consistent with such an integrated approach [8,45]. We believe our data should be interpreted as a candidate pathophysiological framework supported by biomarker profiling, that does not require costly imaging or tertiary infrastructure but would need prospective validation in other cohorts prior to clinical use.

We also point out that the elements of the proposed panel are not to be viewed separately. Nephrinuria is very sensitive but also increases in early reversible glomerular stress [11]; galectin-3 is increased in non-cardiac fibrotic conditions [21,22] and NT-proBNP is affected by renal clearance [20,23,24]. The clinical usefulness of the panel comes from the combined interpretation in the context of the renal etiology and from the inclusion of *E/e′* as a summary of the haemodynamics [19,28].

### 4.2. Strengths and limitations

The strengths of this work are the prospective design, the inclusion of three different etiologies of CKD in the same protocol, the use of an unusually broad panel of biomarkers of podocyte injury, tubulointerstitial fibrosis, myocardial fibrosis and cardiac wall stress in the same patients and the inclusion of stress echocardiography as part of the routine assessment. The retrospective audit is population-based, and the analytical process is in line with the current methodological recommendations.

The limitations are equally important to acknowledge. This was a single-center study and the sample size was sufficient for the planned correlation and ROC analyses but was too small to support multivariable risk-prediction modeling, which we have therefore not attempted to publish, as it would require external validation that we are not yet able to provide. The clinical endpoint was cross-sectional (subclinical cardiorenal syndrome at enrollment) and longitudinal data on hard outcomes (hospitalization, mortality, dialysis initiation) are not yet available; a follow-up phase is ongoing. We did not do cardiac magnetic resonance, which would have been a better platform to anchor the galectin-3 signal directly by imaging myocardial fibrosis [29,38,39]. Patients on dialysis and those with known ischemic heart disease were excluded, by design, which limits generalizability to these patient groups [30,40,46]. Finally, urinary nephrin and type IV collagen ELISAs must be standardized pre-analytically and there is a true inter-laboratory variability that must be taken into account in any future multi-center extension [26,27].

There are a number of additional restrictions that should be stated. First, the study was single center and the sample size was relatively small, limiting the statistical power and external generalizability. Second, no formal *a priori* power calculation was done and some comparisons may have been underpowered. Third, the analyses were unadjusted: no multivariable modeling was performed to adjust for important confounders (such as age, sex, eGFR, albuminuria, blood pressure, diabetes status and concomitant medication including ACE inhibitors/ARBs, SGLT2 inhibitors and diuretics); therefore, the associations reported cannot be considered independent and residual and unmeasured confounding cannot be excluded. Further, detailed medication-use data (such as ACE inhibitors/ARBs, SGLT2 inhibitors, diuretics, beta-blockers and statins) were not systematically collected in this exploratory data set and thus could not be tabulated or adjusted for; a complete medication table is a priority for any validation cohort. Fourth, multiple biomarker and group comparisons were made without multiple testing correction, and nominal *p*-values should be interpreted with caution and the risk of type I error is increased. Fifth, the primary endpoint was based on an operational, research-based definition of a subclinical cardiorenal phenotype, which is not yet externally validated and for which there is no internationally accepted definition. Since this operational phenotype included both renal and echocardiographic variables, incorporation bias cannot be ruled out when assessing the discriminative association of *E/e′* with the same composite endpoint, and the reported AUC values should be considered in this context. Sixth, inter- and intra-observer variability for the post-exercise echocardiographic protocol were not prospectively recorded. Lastly, the observational, mostly cross-sectional design does not allow causal inferences and the lack of validation in an independent cohort should make the results viewed as exploratory and hypothesis-generating.

The incorporation bias is a topic of particular methodological importance. *E/e′* was directly involved in the operational definition of the subclinical cardiorenal phenotype and the same parameter was then tested for its discriminatory ability. Measures of discrimination, like the area under the receiver-operating-characteristic curve, may be somewhat inflated when the variable being measured is part of the reference standard to which it is compared. The reported AUC values should thus be considered exploratory and used with caution and not as validated estimates of diagnostic performance. Future investigations should reevaluate these relationships with outcome measures independent of the biomarker and echocardiographic variables being studied, such as prospectively collected clinical events or an external reference standard, to confirm or disprove the discriminative signal seen here without this circularity.

## 5. Conclusions

This exploratory, single-center study found that an integrated panel of urinary nephrin and type IV collagen, serum galectin-3 and NT-proBNP, and resting and post-exercise echocardiography were associated with an operationally defined early cardiorenal phenotype in CKD patients without symptomatic heart failure. The components of this panel were correlated with each other and with *E/e′,* supporting the notion that subclinical cardiorenal involvement may be a shared renal–cardiac process rather than a coincidence of two organ-specific abnormalities. Post-exercise echocardiography was more discriminatory of this research-defined phenotype than resting imaging (0.81 vs 0.69). In summary, the biomarker panel and echocardiographic parameters were related to the operationally defined early cardiorenal phenotype and should be further validated in larger, multicenter, prospective studies with appropriate multivariable adjustment before any conclusions can be made about their diagnostic value or clinical screening. The six-step model described with the article should be considered a hypothesis-generating conceptual model and not a validated screening algorithm.

## Data Availability

The data presented in this study are available from the corresponding author, upon reasonable request. The data are not publicly available due to privacy and institutional restrictions.
